# Evaluation of a Thiol-Detection Test to Assess Tooth Brushing Efficacy in Dogs

**DOI:** 10.1177/08987564231179898

**Published:** 2023-06-22

**Authors:** Karolina Brunius Enlund, Nadja Rahunen, Sofia Thelander, Lena Olsén

**Affiliations:** Department of Clinical Sciences, 8095 Swedish University of Agricultural Sciences, Uppsala, Sweden

**Keywords:** dental health, calculus, FAS, gingivitis, periodontal disease, dental home care, plaque

## Abstract

Periodontal disease affects more than 80% of dogs over 3 years of age, making it the most common disease in dogs seen in veterinary clinics. Gingivitis, the early-stage of periodontal disease, may be reversible with tooth brushing. Thiol, a sulfuric compound, has previously been shown to correlate with the degree of periodontal disease. In this study, a thiol-detection test was used to investigate daily tooth brushing efficacy in dogs. Twenty-two beagle dogs were subjected to daily tooth brushing for 2 weeks. Gingival index (GI), plaque index (PI), calculus index (CI) and thiol were assessed before treatment (day 1), after 1 week (day 7), after last treatment (day 14), and 2 weeks after treatment finished (day 29). Degree of stress was also assessed using a fear, anxiety and stress (FAS) scale. Both 7 and 14 days of daily tooth brushing showed an improvement in oral health. Thiol decreased significantly and GI and PI improved significantly after 1 and 2 weeks of brushing. No significant improvement in CI was shown. After an additional 2 weeks without brushing, GI and PI had returned to baseline levels. Stress levels decreased from day 1 to day 14. This study suggests that a thiol-detection test can be used to assess tooth brushing efficacy. Tooth brushing has a positive effect on the oral health in dogs as soon as 7 days after commencement.

## Introduction

The most common disease in small animal veterinary practice is periodontal disease, and it is also considered to be under-diagnosed.^1^ Approximately 80% of dogs have signs of periodontal disease after 3 years of age.^2–5^ Untreated periodontal disease can cause discomfort and has also been associated with cardiac, hepatic and renal disease.^6–9^ Periodontal disease is divided into 2 stages, gingivitis and periodontitis. Gingivitis is reversible with dental home care while periodontitis involves tissue loss and is generally considered to be irreversible.^1,10^ Even with tooth brushing, periodontal disease may still develop, just as in humans. Other dental problems, not preventable by tooth brushing, may also be present. Therefore, the recommendation is that dog owners regularly visit a veterinary clinic for examination of their dog’s teeth.^1,11^

Periodontal disease is initiated by dental plaque, a biofilm consisting mainly of bacteria constantly covering the tooth surface in the absence of daily tooth brushing.^12^ During active periodontal disease, thiols are formed by periodontal pathogens.^13^ Thiols are foul-smelling organic sulfur compounds, and a common cause of halitosis.^13^ The thiol-detection test^a^ used in the present study has been shown to correlate with degree of periodontal disease, and it can also detect earlier signs of periodontal disease not detected by visual awake examination.^14,15^ Although anesthesia is necessary for full examination of dental status, signs of periodontal disease (e.g., gingival recession) may be noted in the awake animal. However, disease is often missed, and therefore as a complement a thiol-detection test as a part of the routine wellness appointment is suggested by the American Animal Hospital Association (AAHA)^16^ and the World Small Animal Veterinary Association (WSAVA),^1^ for detection as well as for improved owner communication by owner visualization of periodontal disease. The use of a thiol-detection test has been suggested to enhance client adherence to dental recommendations in veterinary clinics.^17^

Daily tooth brushing is the "gold standard" for dental home care, in dogs as well as in humans. However, in dogs, adherence to the recommendation is low. In fact, less than 4% of dog owners brush their dogs’ teeth every day in Sweden.^18^ In addition, many dog owners report difficulties for the dog to accept tooth brushing.^19^ In humans, a mean plaque reduction of 42% after tooth brushing has been shown by a meta-review.^20^ To the authors’ knowledge, no similar study has investigated the quality of each brushing session, i.e., the mean plaque amount before *versus* after brushing in dogs. However, plaque reduction in dogs may be presumed to be even lower considering the reported difficulty with performing brushing.^19^ Evidence-based approaches to increase both motivation and performance of tooth brushing are therefore needed.^18^ Regular health appointments at the veterinary clinic, information about dental disease and regular follow ups may be ways to improve dental home care in dogs. To achieve high adherence to dental home care recommendations, the cooperation of the dog is imperative. If the dog experiences stress and shows aversive behavior, the owner will likely be less willing to perform tooth brushing as recommended. Reward-based training with positive reinforcement has been shown to be effective for habituation of tooth brushing in a previous study by the authors’ research group.^21^

The aim of this study was to investigate whether a thiol-detection test can be an aid to evaluate the effect of tooth brushing in dogs. A secondary aim was to investigate habituation to tooth brushing in dogs.

## Materials and Methods

The study, including the use of dogs and facilities, was approved by the Ethics Committee for Animal Experimentation, Uppsala, Sweden Approval No: Dnr 5.2.18-7454/15, User permit: Dnr 5.2.18-2636/15.

### Study Design

Tooth brushing was performed once daily on 22 dogs during a 2-week period. Oral health assessment and thiol-detection tests were performed on days 1, 7, 14 and 29. Four dogs were assessed as controls and to ensure blinding of the assessor.

### Study Population

All dogs were housed and used as teaching dogs at the Swedish University of Agricultural Sciences (SLU) in Uppsala, Sweden (Table 1). All dogs lived under the same conditions in groups of 3 to 6 individuals separated according to sex in separate indoor pens with an associated outdoor yard. The dogs had access to rawhide chews^b^ and free access to water. Dogs were fed one of two types of dry food^c,d^ twice daily. As training rewards, all dogs received similar amounts of treats^e^. The dogs were otherwise treated according to current routines for the university. Fifteen of the participating dogs had in the past (3 months to several years prior to the study) been subjected to a thorough dental examination and dental cleaning under general anesthesia, and none showed signs of generalized periodontitis.

**Table 1.** Sex and Age of Participating Dogs.

Dog no.	Sex	Age (years)	Dog no.	Sex	Age (years)	Dog no.	Sex	Age (years)
Dog 1	Female	4	Dog 12	Female	4	Control dog 23	Female	4
Dog 2	Female	4	Dog 13	Female	6	Control dog 24	Female	11
Dog 3	Female	4	Dog 14	Female	4	Control dog 25	Male	11
Dog 4	Female	4	Dog 15	Female	4	Control dog 26	Male	6
Dog 5	Female	4	Dog 16	Female	4			
Dog 6	Female	11	Dog 17	Female	4			
Dog 7	Female	12	Dog 18	Male	3			
Dog 8	Female	4	Dog 19	Male	8			
Dog 9	Female	11	Dog 20	Male	3			
Dog 10	Female	4	Dog 21	Male	8			
Dog 11	Female	11	Dog 22	Male	6			

### Tooth Brushing

Tooth brushing was performed once daily during a 2-week period. Each dog was assigned an individual toothbrush^f^. The dogs were rewarded verbally, tactilely and with treats in conjunction with brushing with toothpaste^g^. Tooth brushing was performed by same authors (NR/ST), who brushed every other dog in random order. The toothbrush was tilted about 45 degrees towards the tooth and gingiva and moved in circular motions for 1 minute on each side on the buccal surfaces.^22^ Control dogs were not brushed.

### Assessment of Dental Health

**Figure 1.**
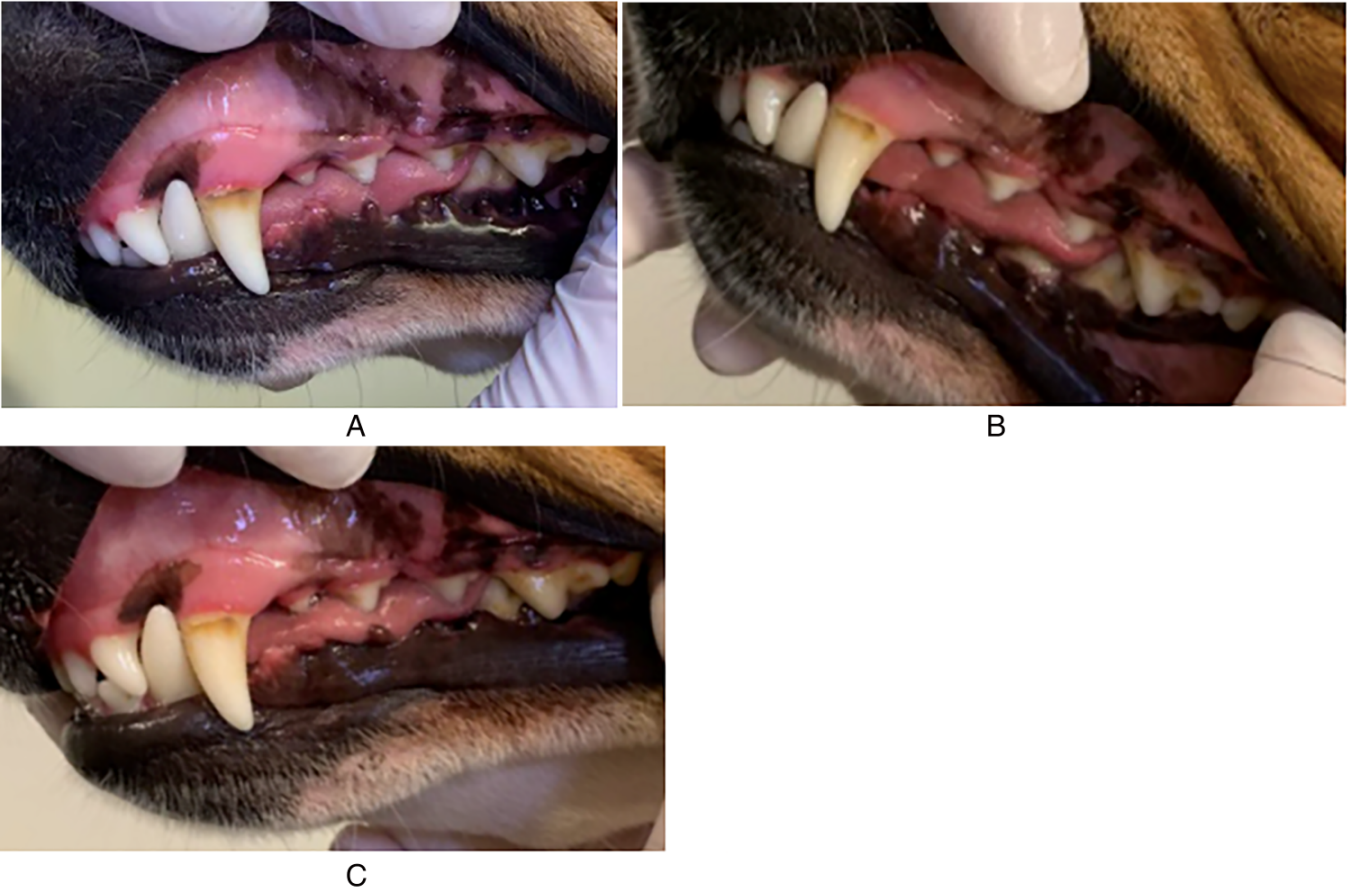
Case # 20. The left maxillary teeth were assessed. (A) Day 1 was scored as gingival index (GI) - 2 and calculus index (CI) - 2. (B) Day 14. GI - 0 and CI - 2. (C) Day 29. GI - 1 and CI - 2.

The assessment was performed by the same author (KBE). All dogs were examined on an examination table in the same room. The buccal side of the maxillary third incisor, canine and first, second, third and fourth premolar teeth (I3, C1, PM1, PM2, PM3, and PM4) were assessed without sedation or anesthesia on days 1, 7, 14 and 29. The protocol used assessed gingival health^23^ and plaque and calculus^24^ according to previous published studies (Table 2, Figure 1A-C). Bleeding on probing was not assessed.

**Table 2.** Dental Health Assessment Protocol: Gingival Index (GI),^23^ Plaque Index (PI),^24^ and Calculus Index (CI).^24^

**Gingival index (GI)**	
0	No inflammation
1	Mild inflammation, mild hyperemia
2	Moderate inflammation, moderate hyperemia
3	Severe inflammation, severe hyperemia, swelling, bleeding spontaneously, ulceration
**Plaque index (PI)**	
0	No plaque
1	Thin layer of plaque along the gingival edge
2	Moderate layer of plaque and/or plaque in sulcus
3	Abundant plaque and soft material in sulcus
**Calculus index (CI)**	
0	No calculus
1	Supragingival or calculus that extends only slightly below the free gingival margin
2	Moderate amount of supra- and/or subgingival calculus or only subgingival calculus
3	Abundant supragingival and/or subgingival calculus

**Figure 2.**
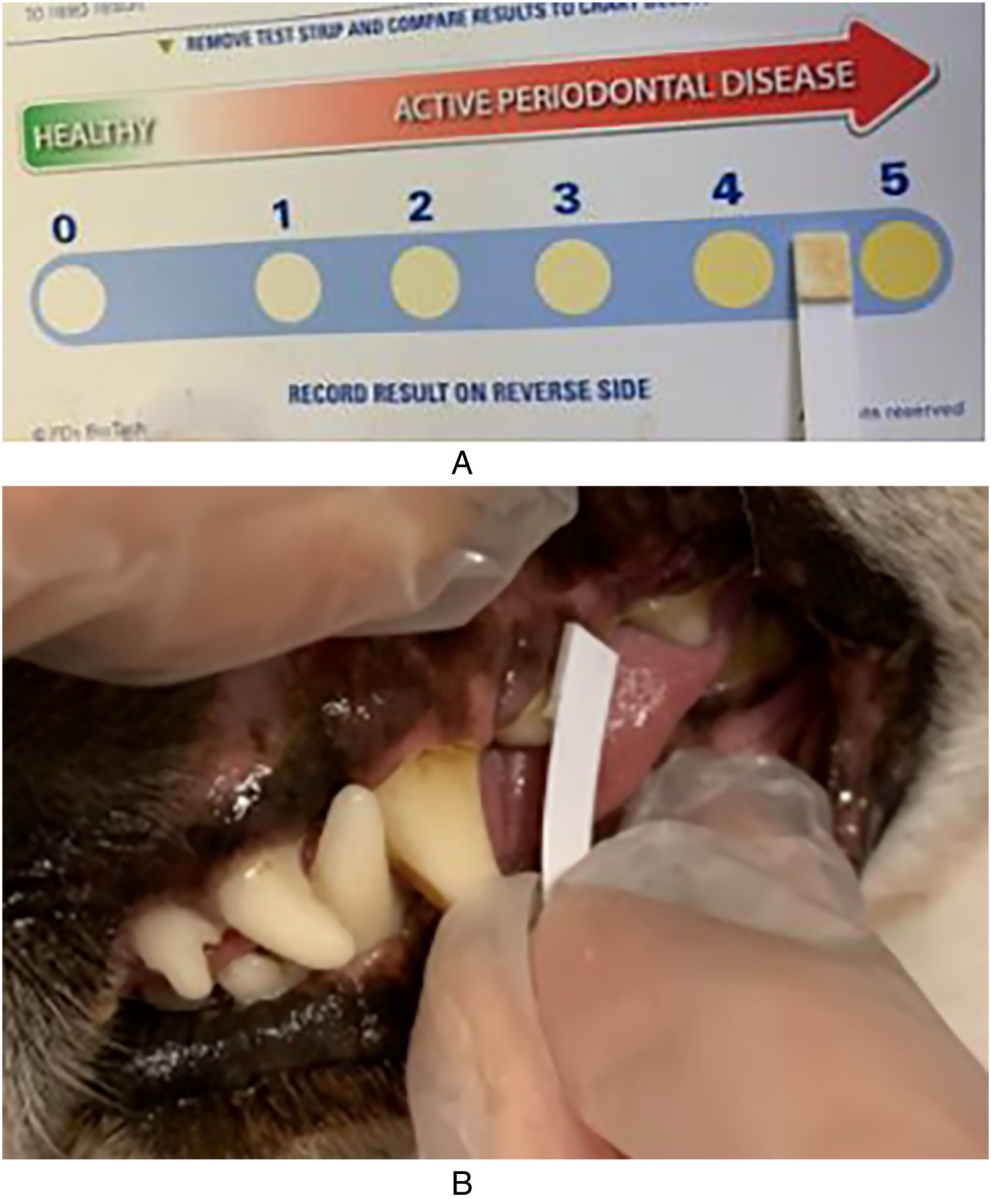
The thiol-detection test. (A) Scale used in the visual assessment of the thiol content in the dogs’ mouths on day 1, day 7, day 14, and day 29. (B) The thiol-detection test was used by gently gliding the pad of the test strip along the entire maxillary buccal gingival margin for 5-10 s.

In addition to the assessment protocol, a thiol-detection test was used according to the manufacturer's instructions by gently gliding the pad of the test strip along the entire maxillary buccal gingival margin for 5 to 10 s. The thiol-detection test was read according to the instructions after another ten seconds and graded by the assessor according to the manufacturer's scale of 0 to 5. All test strips were assessed in room lighting (i.e., fluorescent lamp in ceiling and indirect window light) (Figure 2A and B).

### Fear, Anxiety and Stress (FAS) Assessment

One way to measure fear, anxiety and stress (FAS) in dogs is by the use of a FAS scale, where low FAS is assessed as level 0 to 1, moderate FAS is assessed as level 2 to 3, and high FAS is assessed as level 4 to 5.^25^ At level 1, mild signs of stress are seen, e.g., licking around the mouth, avoiding eye contact, or lifting the paw. At level 2, signs are seen more often, and may include that the ears are angled slightly backwards, tail hangs down, or the dog may be overly attention-seeking. At level 3, even more signs are seen, and they are shown more often, the signs are the same as level 2, but the dog may refrain from receiving treats, or take it carefully. The dog may also be hesitant to interact with humans. Level 4 shows severe signs of stress or fear such as trying to escape, excessive panting, tense closed mouth, ears angled backwards, and tail between legs. At level 5, most signs of stress or fear are shown, including aggression. Signs of aggression can be lunging, barking, growling, or biting.^25^

Every day during tooth brushing, the dogs’ stress levels were assessed and documented according to a FAS protocol.^25^ FAS level 4 was chosen a priori as the breaking point, meaning that the activity would be interrupted if any dog showed signs of FAS level 4 or higher. Nineteen of the 22 included dogs had received dental care previously, with toothbrush or textiles, 1 year prior to this study, where FAS was assessed in a similar manner.^21^ Control dogs were not assessed for FAS.

### Statistical Analysis

To compare before *versus* after treatment values of gingival index (GI), plaque index (PI), calculus index (CI), and thiol-detection test, values were analyzed pairwise (intervention day *versus* baseline), by a one-sided Wilcoxon signed rank test. The significance level was set at *P* < 0.05. Pearson and polychoric correlations were calculated between the thiol-test, GI and PI. CI was not considered for correlation analysis since it did not change significantly in the Wilcoxon test. Wilcoxon tests and correlation analyses were performed in R v 4.0.5. Mean and standard deviation for GI, PI and CI, as well as thiol-test values and FAS-values, were calculated using Excel.

## Results

### Assessment of Dental Health

**Figure 3.**
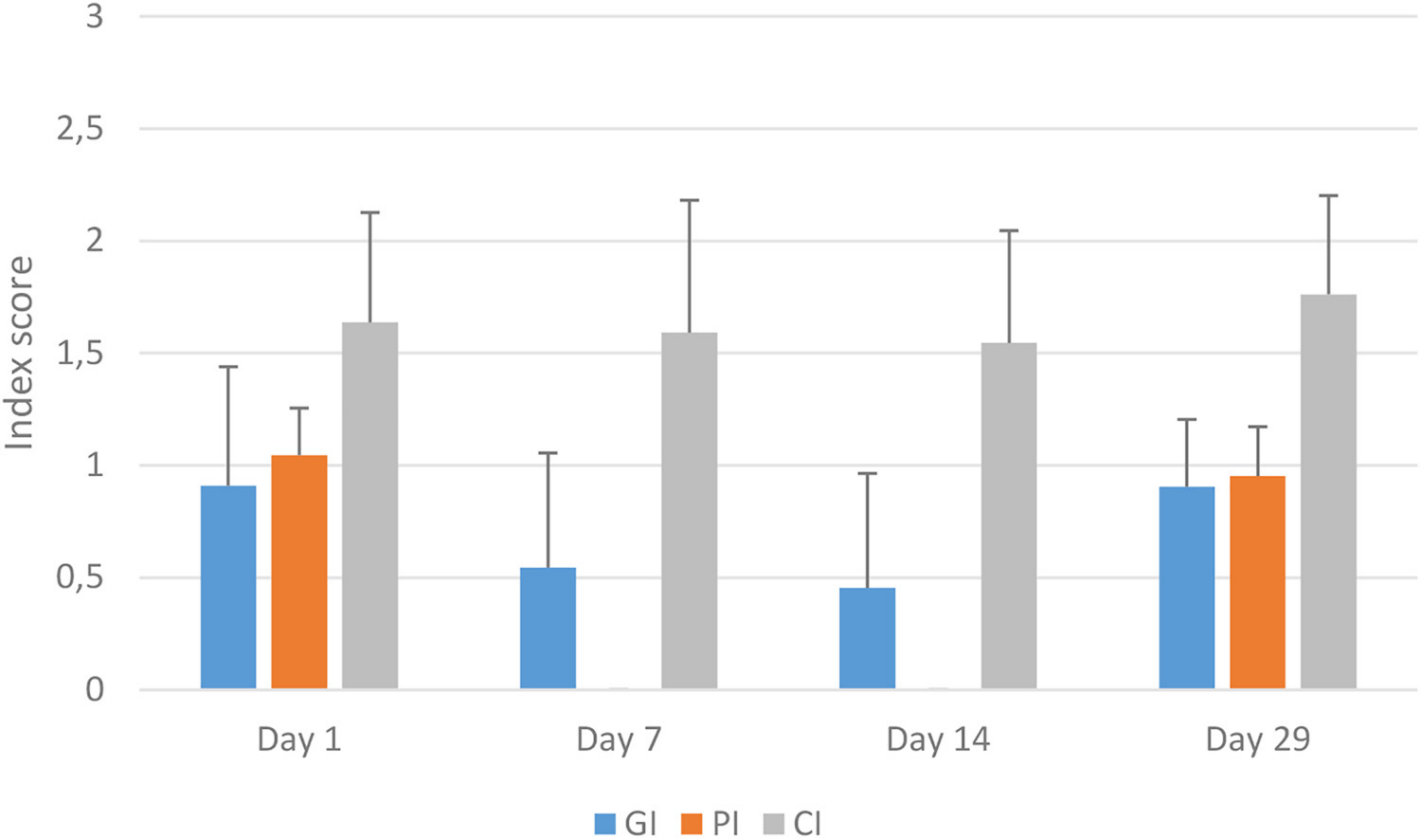
Mean values with standard deviation for gingival index (GI), plaque index (PI), calculus index (CI). GI, PI and CI assessed on a scale 0-3.

Both 7 and 14 days of daily tooth brushing showed an improvement in oral health. GI improved after 7 days of daily brushing (*P* < 0.006) and 14 days of tooth brushing (*P* < 0.005). PI also improved after 7 and 14 days (*P* < 0.005) and no dog was assessed as having remaining plaque after brushing. No significant difference in CI was shown after 7 or 14 days of tooth brushing, or after discontinuing tooth brushing by day 29. Day 29, after 2 weeks without brushing, GI and PI had returned to baseline levels (day 1) (Figure 3).

**Figure 4.**
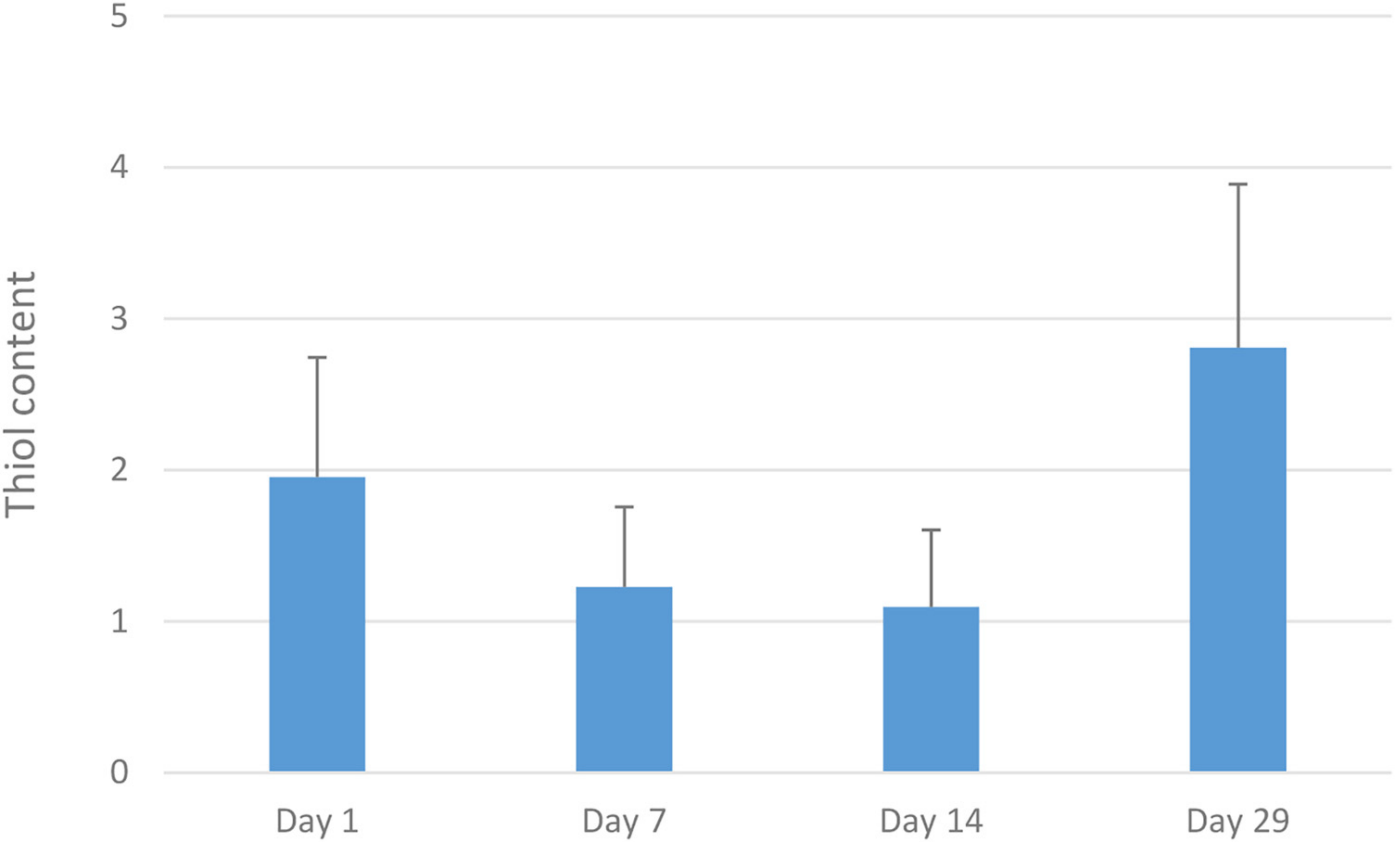
Mean values with standard deviation for thiol-detection test values, assessed on a scale of 0-5.

**Figure 5.**
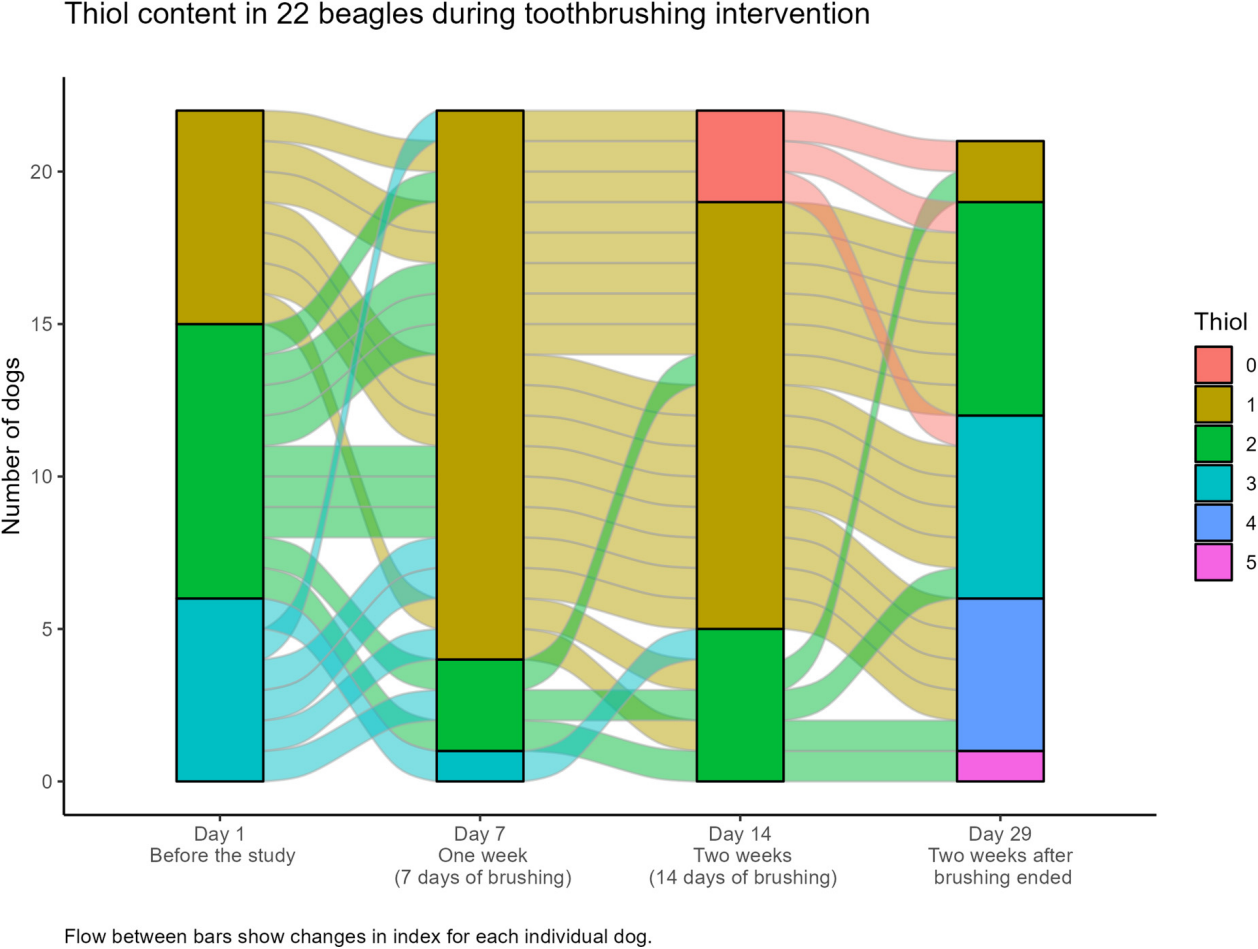
Flow chart of the individual Beagle dog's (*n*  =  22) thiol-test on days 1, 7, 14, and 29. Flow between bars shows changes in thiol-detection test result for each individual dog.

In addition, both 7 and 14 days of daily tooth brushing showed the thiol value (*P* < 0.002) to have been reduced. Thiol measurement day 29 (i.e., after 2 weeks without brushing) was higher than on day 14 (*P*  =  0.0001), and also higher than on day 1 (*P*  =  0.005) (Figures 4 and 5).

The thiol-detection test correlated modestly with visual assessment of the dogs’ GI (*r*_Polychoric_  =  0.35; *r*_Pearson_  =  0.27) and more strongly with PI (*r*_Polychoric_  =  0.75; *r*_Pearson_  =  0.61). In addition, GI and PI correlated at an intermediate level (*r*_Polychoric_  =  0.53; *r*_Pearson_  =  0.36).

The control group was primarily used to ensure blinding of the assessor. However, although the control group was small, both PI and thiol-test values were significantly lower in the treatment than in the control group, after 7 and 14 days of brushing (*P* < 0.003).

### Fear, Anxiety and Stress (FAS) Assessment

**Figure 6.**
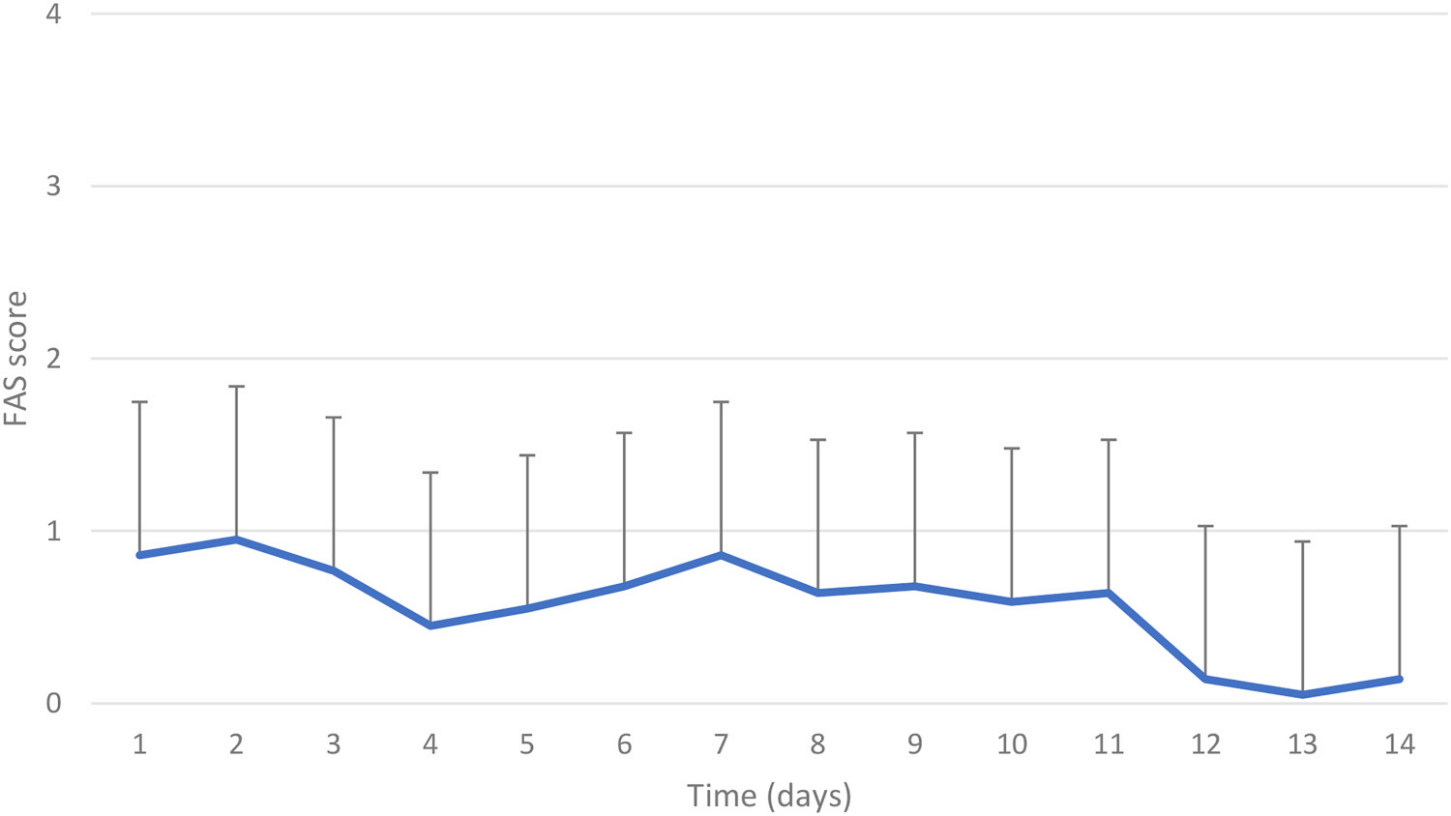
Mean FAS score (grade 0-5) with standard deviation for days 1 to 14 in 22 beagle dogs during tooth brushing.

The dogs showed fewer signs of stress on the last day compared to the first day (Figure 6). The mean FAS level at day 7 (0.9  ±  0.6) (mean  ±  SD) was not lower than at day 1 (0.9  ±  0.9). However, FAS at day 14 had decreased to 0.1  ±  0.4 (*P* < 0.004).

## Discussion

### Dental Health

For a thorough dental examination, dogs require anesthesia. In addition, a professional dental cleaning is recommended before initiating tooth brushing, so teeth will be clean and any problems treated.^10^ However, in some cases this may not be achievable, and tooth brushing may still be recommended because of its benefits. In the present study, the authors observed that tooth brushing improved oral health by 7 days. In fact, all dogs had reduced their PI to 0 regardless of the starting level. The results from this study also indicate an effect of tooth brushing on gingivitis within 7 days, although previous studies have shown that a complete return from gingivitis to healthy gingiva may require 6 weeks of brushing twice daily,^26^ or 24 weeks of daily brushing.^27^

After 2 weeks of not brushing, plaque had returned in all but one of the dogs, and GI had also returned to its baseline level, highlighting that continuous tooth brushing is required to maintain good oral health, in accordance with previous studies.^28–30^ The rapid deterioration of dental health when tooth brushing stopped for 14 days may also serve as a motivation for pet owners to continue tooth brushing.

Even though CI was not significantly affected by 2 weeks of tooth brushing, calculus had in some dogs disappeared along the gingival margin, while still present coronally (grade 2). Although calculus is generally considered too dense to brush away completely, this observation indicates that a reduction of calculus may be possible with daily tooth brushing, in accordance with a previous study from this research group.^21^

Normally, the teeth of the dogs in this study are not brushed at all. They always have access to rawhide chews yet still develop calculus. The present study thus supports previous research that tooth brushing is more effective for dental health than is passive dental care such as use of chews.^1,31^

### Thiol-Detection Test

Periodontal disease is commonly underestimated by awake examinations and tools for early detection may play an important role, especially in communication with pet owners regarding the need for intervention. A previous study showed that a thiol-detection test is more sensitive than a visual awake examination.^14^ This is supported by the present study, where 4 dogs had low values in the visual assessment at day 1 (GI  =  0, PI  =  1) while the values were higher according to the measurement of thiol (grade 2-3).

The thiol-detection test is recommended for use in the veterinary clinic for pet owner visualization of periodontal disease and in order to detect early signs of disease.^16,17^ In the present study, the authors show that the thiol-test yields lower values after initiation of tooth brushing. It could therefore be suggested that the thiol-detection test strip may be used in a home environment as a way to monitor performance of dental home care. The thiol-detection test may indicate whether tooth brushing needs to be improved either in frequency or quality of performance. The authors also propose its usefulness as a complement to other dental assessment variables in research settings where a full dental examination under anesthesia is not an option. However, more studies are needed, with inclusion of dogs diagnosed with periodontitis.

### Dental Home Care and FAS

A previous study has shown that although 29% of dog owners consider tooth brushing very important for dental health, only 4% brush daily, and it is common for dogs to be uncooperative when owners attempt to brush.^18^ Another study showed that as many as 20% discontinued tooth brushing because the dog did not cooperate.^32^ In the present study dogs showed a lower stress level after 2 weeks of daily brushing, indicating the potential to train dogs to accept dental home care procedures. The authors propose that veterinary clinics regularly offer appointments to provide training opportunities for animal owners in tooth brushing technique, and discussions about dental health. This practice is in line with that of human dentistry, with regular visits to the dental hygienist and dentist to follow up on dental home care and discuss dental home care practices and techniques.

The dogs in this study are not usually subjected to daily tooth brushing. Most of them were, however, part of a different study (1 year previous to the present study) which included 5 weeks of daily active dental home care, with textiles or tooth brushing.^21^ Therefore they had some previous experience with active dental home care which may explain the relatively low FAS baseline in this study.^21^ Nevertheless, the stress levels showed that it took longer than 7 days to get the dogs accustomed to handling and tooth brushing. However, mean stress levels had decreased considerably by day 14. In fact, dogs with higher stress levels, FAS 2 (*n*  =  6) and FAS 3 (*n*  =  1), lowered their FAS down to 1. The habituation seen in the dogs to the tooth brushing procedure during the study can be a motivation for dog owners to continue practicing, with positive reinforcement such as praise and treats, to brush their dog's teeth at home.

### Methodological Considerations

The protocol used for assessment of dental health was adapted for examination of fully conscious dogs, without sedation or anesthesia. The degree of periodontal disease is therefore likely underestimated.^1^ Moreover, beagle dogs were used in the present study, whereas other breeds may differ in dental health and stress susceptibility. Also, FAS levels were assessed by the authors (NR/ST) who also performed the tooth brushing, whereas blinded FAS assessment by an independent part may have produced different results.

Professional dental cleanings in study dogs were performed from several months to several years prior to the study, and thus had minor effect. All dogs had some degree of calculus and plaque at the beginning of the study, regardless of dental cleaning history, and the longitudinal design ensured that each dog acted as its own control. Also, 2 different diets were fed, however, the primary analysis was that of before-*versus*-after and not *versus* control group, again ensuring that each dog acted as its own control.

The control group consisting of 4 dogs was used primarily for blinding the assessor. Although the group was small, the difference in PI and thiol-test compared to the intervention dogs increases the validity of the results. In addition, the validity of the thiol-test to assess dental health was enhanced by correlations with PI and GI.

Thiol measurements were, surprisingly, higher on day 29 than on day 1. It is unlikely that dental health would deteriorate over the time span in question, and the reason for this observation is unknown. However, the assessment of the strips is subjective, and this result may indicate a lower-than-desired intra-rater reliability. The authors observed that the test result could be interpreted differently depending on the lighting (room/window/examination light), and some intra-rater bias may be in place due to incomplete blinding, since the assessor knew what day of intervention the assessment took place. Also, in some thiol measurements contamination of the test-strip could not be excluded, potentially affecting assessment. The manufacturer’s instructions warn that contamination such as blood can give a false value. Future studies should thus include an assessment of inter- as well as intra-rater reliability as well as the influence of technical factors. However, the possibility needs also to be considered that the high thiol levels 2 weeks after the end of intervention could reflect for example a shift in plaque microbiota composition and/or activity. In fact, it was previously reported that several bacterial phyla, suggested as canine periodontal pathogens, actually increased in relative abundance 1- and 2-week post prophylactic dental cleaning.^30^

## Conclusion

The thiol-detection test showed a significant reduction after initiation of tooth brushing, and a concomitant reduction in GI and PI. These levels also increased notably 2 weeks after termination of brushing, showing that the thiol-test may be used as a measure of active dental home care in the form of daily tooth brushing. The test could consequently be used in veterinary clinics as grounds for discussion with pet owners about improved dental home care, or to show improvement after initiation of daily tooth brushing.

## Materials

- OraStripdx, Pdx Biotech LLC, Washington, USA
- Dogman AB, Åstorp, Sweden
- Purina Pro Plan Veterinary Diets Hypoallergenic, Nestlé, Switzerland
- Royal Canin Veterinary Diets Dog Gastrointestinal, Mars Incorporated, USA
- Purina Pro Plan Veterinary Diets Canine HA Hypoallergenic, Nestlé, Switzerland
- Kids soft, Colgate-Palmolive, USA
- DogaNova, Petsosan, Bergen, Norway
